# The climate *battles of ideas*: Minority discourses in readers’ comments to climate change articles in the Portuguese press

**DOI:** 10.1177/09636625241254505

**Published:** 2024-06-12

**Authors:** Nuno Monteiro Ramos, Paula Castro

**Affiliations:** Instituto Universitário de Lisboa (ISCTE-IUL), Portugal

**Keywords:** battles of ideas, climate change minority discourses, meaning barriers, public understanding of climate change, readers’ comments, techniques of neutralization

## Abstract

Today, the dominant climate change discourses affirm its anthropogenic nature and the urgency for policies. However, minority discourses remain active in the worldwide debate, refining arguments beyond simple denial—as shown regarding formal/official discourses of the United States and European far-right parties. This makes it necessary to examine the public understanding of climate change in everyday, informal minority discourses, looking at how they work for broadening societal space for “quarantining” the transformative potential of climate change meanings/policies. For this, we analyze readers’ comments on climate change articles from two Portuguese newspapers, drawing from the frameworks of neutralization techniques and meaning barriers. Findings show that although denial of anthropogenic climate change remains, discursive efforts concentrate on person-stigmatizing depictions of climate change actors, delegitimized as “elites” in populist vocabularies, reflecting a consistent alignment between everyday discourses and those of the United States and European official far-right. We discuss the functions this pattern may have for the growth of climate change minority positions.

Today, the dominant discourses of climate change (CC) affirm its anthropogenic nature, harmful consequences, and the urgent need for CC policies, and these are facts supported by a worldwide scientific consensus (Hornsey and Fielding, 2020; IPCC, 2021; Powell, 2019). Yet alternative, minority CC discourses continue to exist (Boykoff, 2016; Lamb et al., 2020; Lockwood, 2018; McKie, 2018). Some still directly deny the existence and/or the anthropogenic nature of CC, expressing *epistemic* skepticism; others develop *response* skepticism (Capstick and Pidgeon, 2014), questioning CC policy and/or action with ever-evolving combinations of arguments (Forchtner and Lubarda, 2023; Lamb et al., 2020; McKie, 2018).

The study of how these minority discourses work and intervene in the worldwide CC debate contributes to a better understanding of what the psychosocial approach of social representations calls *battles of ideas* (Castro et al., 2018; Magioglou and Coen, 2021; Moscovici and Markovà, 2000): how social groups with different interests construct their meanings and struggle to spread and impose them, and to resist the meanings of others. In these *battles*, or struggles for meaning, different views of what is acceptable/unacceptable, possible/impossible for tackling CC confront each other (Carvalho et al., 2021; Castro et al., 2018; Hochachka, 2021; Magioglou and Coen, 2021), with some achieving dominance in shaping individual, collective and institutional responses (Castro and Mouro, 2016; Hulme, 2020). Dominance over meaning is, however, always unstable and threatened, and minority views can in time spread and grow—in both formal/institutional contexts and informal/everyday contexts (Castro and Mouro, 2016; Hochachka, 2021; Magioglou and Coen, 2021). This makes it crucial to better understand how the CC struggles for meaning are waged from non-dominant/minority discourses and how they move between contexts.

Regarding formal/institutional contexts, research has lately evidenced how CC minority official discourses—made in the United States by the *Climate Change Countermovement*, a network of right-wing corporate and political actors (Almiron et al., 2020; Boykoff, 2016; Dunlap, 2013), and in the EU parliament by the right-wing and populist parties (Forchtner and Lubarda, 2023)—are converging. Today, they focus less on *epistemic skepticism* and more on contesting CC policies, namely contrasting “the people” with a “cosmopolitan CC elite” (Forchtner and Lubarda, 2023; Lockwood, 2018; McKie, 2018).

These CC minority discourses can also be found in the mainstream media—but in Europe less than previously (Domínguez et al., 2016). Importantly, however, today, they are present in online readers’ comments—a new hybrid forum where public understanding of CC meets the more formal CC press coverage, increasingly relevant for understanding today’s CC *battles of ideas* (Coen et al., 2021; Crawford et al., 2019; Jaspal et al., 2013; Walter et al., 2018). Considering the several recent electoral successes of EU right-wing parties associated with CC minority discourses, more research is now necessary to establish *to what extent* their CC arguments *are being reproduced in* reader’s comments. One country deserving attention is Portugal, where such parties went from having two parliamentarian seats in 2019 to having 20 in 2022. Therefore, online readers’ minority comments on CC press articles are the focus of this study.

The comments will be explored with a constructionist psychosocial perspective with two main assumptions (Batel and Castro, 2018; Dixon, 2017). The first is that meanings about the world, coalescing in worldviews, are jointly created through discourse, communication, and other social practices; through them, they are also made available to be learned and reproduced; however, meanings are received and interpreted by agentic individuals able to also contest and/or re-construct them in new, transformative ways (Hall, 1993; Moscovici and Markovà, 2000). The second is that the relative position of different discourses—that is, how dominant or minoritarian in the global debate are the worldviews they convey—matters for developing a better understanding of their functions in defending social positions and worldviews, “quarantining” their meanings from change, and for understanding how they spread to other contexts (Batel and Castro, 2018; Castro et al., 2018; Dixon, 2017; Gillespie, 2020; Jaspal et al., 2013).

In this context, our goal is to analyze readers’ comments to CC-related articles in two Portuguese mainstream newspapers of different political orientations to shed further light on public understanding of CC in everyday minority discourses in Portugal and how it connects to the wider CC debate. Following Moscovici (1994: 238), we adopt a social-psychological definition of minorities as groups of people that think, talk, and act outside the norm, deviating from most members of the community.

Our analysis will (1) contribute to revealing the extent to which CC minority arguments are present in the mainstream press readers’ comments; it will (2) explore how substantive comments with well-articulated minority arguments organize their discursive efforts in making sense of CC and posing barriers to dominant meanings; for this we draw from the Neutralization framework (Sykes and Matza, 1957), already employed to analyze the arguments of the US CC Countermovement (McKie, 2018) and from the literature on semantic and meaning barriers (Castro and Santos, 2020; Gillespie, 2008, 2020; Obradović and Draper, 2022; Uzelgun et al., 2016), examining how three barriers—avoiding, delegitimizing and limiting (Gillespie, 2020)—are mobilized for “quarantining” minority meanings from dominant ones. (3) Overall, this will allow a discussion of the similarity of the discursive efforts of our comments to those of the United States and European far-right parties, relevant because mobility in meaning is an indication of mobility in power relations and provides a broader consideration of how battles of ideas are/can be waged from minority positions.

In what follows, we first present an overview of research on CC minority positions across nations, and in the press, and of how neutralization theory and the study of meaning barriers can contribute to a better understanding of the CC debate. Afterwards, the methodological and analytical decisions are presented. Finally, results are reported and discussed.

## 1. Climate change’s minority positions across nations

In 2019, 13% of the Americans polled in a 23-country survey agreed that the climate was changing but disagreed that the changes had anthropogenic origins, as did Saudi Arabians (16%) and Indonesians (18%) (Milman and Harvey, 2019). Meanwhile, such skepticism was almost residual in China, the United Kingdom, and EU (Milman and Harvey, 2019). This might suggest that CC minority positions are residual in the worldwide CC debate. Yet, the rejection of anthropogenic CC is only one dimension of CC contestation. Some discourses advance instead *response* skepticism (Capstick and Pidgeon, 2014), accentuating for instance the economic downsides of CC policies or how CC action is impossible without a global population reduction (Lamb et al., 2020).

In the United States, research has revealed that right-wing positioning is the most important factor in predicting CC minority positions (Hornsey et al., 2018). This may be because, in the United States, this link has long been re-enforced by a well-organized conservative “denial machine” (Dunlap, 2013: 692) aligned with the interests of the fossil-fuel industry (Dunlap and McCright, 2015), the CC Countermovement. Nonetheless, today US arguments are making their way into European contrarian think-tanks (Almiron et al., 2020). Moreover, similar efforts can be detected in Europe, as Nordic and German far-right and populist parties’ discourses now attack “elites,” defending a climate nationalism for protecting *our* land (Vihma et al., 2021) and the *core people* (Küppers, 2024), and Western European right-wing parties contest the EU’s supranational CC policies embracing nationalist discourses (Huber et al., 2022; Lockwood, 2018; Kulin et al., 2021). Furthermore, it is now evident that the far-right argumentation in the European Parliament changed over time, with simple denialism, or *epistemic* skepticism, fading and opposition to climate policies, or *response* skepticism, rising (Forchtner and Lubarda, 2023: 63). It is thus now important to focus on everyday CC minority discourses in Europe to understand to what extent—and how—they are spreading the updated meanings and arguments of formal forums, making them available for publics (and voters) to learn how to defend them from majority ones. One way to do this is by focusing on readers’ comments to CC press articles.

## 2. Media engagement in the climate change debate

The mainstream media is a crucial factor in the struggles for meaning about CC, with the works of journalists and opinion-makers contributing to the joint production and transformation of meanings regarding CC (Boykoff and Boykoff, 2004; Carvalho, 2007; Crawford et al., 2019; Jaspal et al., 2013). In the past, CC minority positions in the media have been found to result, in part—although not always (Jaspal et al., 2013)—from the journalistic norm of balance (Boykoff and Boykoff, 2004): that is, the demand to present in a balanced way the opposing/different positions on a given subject, regardless of their scientific status or social consensus. Today, nonetheless, articles contesting CC seem to be much less visible in the European mainstream press (Domínguez et al., 2016).

However, the Internet brought new complexities to the CC *battles of ideas* in the press, enabling readers to contribute to the debate through the comments sections—interactive platforms where everyday/public understandings of CC and media coverage of CC sometimes clash (Boykoff and Boykoff, 2004; Jaspal et al., 2013; O’Neill and Boykoff, 2010; Walter et al., 2018). In these everyday forums, some readers’ comments construct an oppositional reception (Hall, 1993) of the published pieces that convey affirming CC views; the comments consequently contribute to keep minority CC views alive despite the meanings made available in the pieces themselves (Jaspal et al., 2013; O’Neill and Boykoff, 2010; Walter et al., 2018). Offering anonymity, these informal forums shield users from the risk of social isolation when they offer oppositional readings expressing minority worldviews (Walter et al., 2018), contributing to the formation of “Internet bubbles” or “echo chambers,” reinforcing pre-existing views more than promoting debate (Hornsey and Fielding, 2020; Walter et al., 2018).

In this regard, studies in non-mainstream press venues (e.g., tabloids) have already shown how CC articles expressing minority views are popular targets for readers’ supportive comments (Walter et al., 2018). The readers’ reception thus provides “echo chambers” for the skeptic, minority worldviews offered in the news coverage of such outlets (Boykoff and Mansfield, 2008; Walter et al., 2018). It is now crucial to study the comments to CC pieces also in the mainstream press of different orientations, as a way to better understand their reception, and whether and how they are reproducing the discursive efforts found in formal/political CC minority discourses.

## 3. Making sense of climate change: Techniques of neutralization and meaning barriers

As mentioned, in adapting to the changing worldwide debate, the two generic CC minority discourses of *epistemic* and *response* skepticism also acquire updated shades (Fonseca and Castro, 2022; Forchtner and Lubarda, 2023; Uzelgun et al., 2016). Therefore, nuanced ways of examining their contents and functions are necessary. Because they are minority meanings, one entry point for this is to treat them as discourses deviating from, or violating, the culturally dominant norm, which makes the theoretical framework of the *Techniques of Neutralization* (Sykes and Matza, 1957) a suitable approach.

This framework was developed to study the discursive strategies employed for justifying non-normative or deviant behavior in juvenile delinquency, avoiding self and other condemnation (Sykes and Matza, 1957). However, research with the framework has since expanded beyond this realm, and meanwhile, overlapping concepts—such as disclaimers (Hewitt and Stokes, 1975), or cognitive mechanisms of moral disengagement (Bandura, 2007)—were developed for identifying categories of arguments people recurrently employ when they recognize the existence of (majority) norms, but still violate them (see Kaptein and Van Helvoort, 2019; Maruna and Copes, 2005; McKie, 2018). Under this reasoning, the five categories of the Neutralization framework have directly been adapted for studying changes in discourses of the US CC Countermovement organizations over time (McKie, 2018), The adapted categories are as follows: (1) Denial of (Human) Responsibility—CC exists, but humans are not the cause; (2) Denial of Injury—human action is not causing any significant CC-related harm; (3) Denial of Victim—there are no CC victims, and if there are, they deserve to be victimized; (4) Condemnation of the Condemner—CC research is misrepresented by scientists, and manipulated by media, politicians, and environmentalists; (5) Appeal to Higher Loyalties—economic progress is more important than preventing CC.

Findings show that the discursive efforts of the US CC Countermovement organizations indeed changed in time, with the use of simple CC denial categories (or epistemic skepticism) decreasing, with that of categories contesting and condemning CC protagonists as manipulative or wrong increasing (Levy and Egan, 2003; McKie, 2018), helping protect fossil-fuel based economies (Dunlap and McCright, 2015).

The functions these categories perform can be better understood by looking also at the *meaning barriers* they include (Gillespie, 2008, 2020). From the psychosocial tenet that the joint construction of meaning also joins people together within shared worldviews (Batel and Castro, 2018; Obradović and Draper, 2022), these “barriers” are conceptualized as the discursive and argumentative tactics used to defend our worldviews against alternative ones (Gillespie, 2008), avoiding changing one’s mind in the *battles of ideas* (Obradović and Draper, 2022). Three layers of defense can be distinguished: (1) *Avoiding*—when our arguments ignore or deny the meanings of others; here we can assume that the *Neutralization* categories useful for *avoiding* are those denying that CC is human-made, makes victims, or bring harms; (2) *Delegitimizing*—when arguments delegitimize the others and their meanings, stereotyping and stigmatizing them, depicting their meanings as false (see Obradović and Draper, 2022); the category Condemnation of the Condemner can work for these functions; (3) *Limiting*—when discourse attempts to restrict the impact of certain meanings, for example by defending that if implemented they would “risk undermining another valued goals” (Gillespie, 2020: 23), a category with parallels to Appeal to Higher Loyalties.

One of the goals of this study is to explore how minority argumentation in readers’ comments works in posing barriers to dominant, CC-affirming meanings. This means focusing on the functions comments themselves may perform at the societal level, extending approaches that focus more on their personal functions—for example, how they help construct moral disengagement for the person (Bandura, 2007). This implies seeking to understand how the arguments the comments make available to be learned and reproduced by others are broadening societal space and power for CC minority worldviews and the different ways in which they attempt to “quarantine” the transformative potential of CC-affirming ideas. In this regard, the analysis will explore whether (and how) comments (1) privilege full *avoidance* of dominant meanings, a confrontational choice closing down negotiation (Castro and Santos, 2020); (2) focus on undermining dominant views and actors; (3) or tread the less confrontational path of trying to *limit the effects of* dominant meanings by recognizing merits in opposing meanings before identifying other valuable goals they negatively affect (Castro and Mouro, 2016; Uzelgun et al., 2016).

## 4. Method

### Newspapers and period selection

We collected comments to CC-related articles in two mainstream Portuguese online newspapers: Diário de Notícias (DN) (www.dn.pt) and Observador (www.observador.pt). The newspapers were selected according to a combination of three criteria: (1) different editorial stances: DN (2015) assumes a politically moderate viewpoint, whereas Observador (2014) supports a right-wing viewpoint; (2) high popularity: DN is one of the two more widely-read daily reference newspapers in Portugal and Observador also has high popularity (APCT, 2022); (3) having online versions with the possibility to comment, where the policy for comments is similar: free sharing of opinions by commentators is allowed and filtered only to maintain civility.

We investigated a two-and-a-half-year period: from 1 January 2019 to 30 June 2021. This means that the period covered includes one year before COVID-19 (2019), and one and a half years during COVID-19. The year of 2019 was ideal for focusing on the climate debate, as it was a particularly noteworthy year for environmental activism and calls for CC action, including the “biggest climate protest ever” (Guardian, 2019), and the rise to world-fame of the Swedish activist Greta Thunberg. In turn, 2020 and 2021—the years of COVID-19’s outbreak—were relevant as years of tension in science-society relations, leading to the re-discussion of hegemonic representations of science, politics/democracy and nature (Magioglou and Coen, 2021). Furthermore, these were years for which it was anticipated that people would be especially involved in online activities, including press comments, because of the exceptional circumstances of remote working and lockdown. A large number of comments on CC-related articles from the two-and-a-half-year period in both newspapers was indeed found, and we then decided to stop collecting articles and comments in June 2021.

### Search and collection of articles and comments

DN and Observador have different ways of archiving data on their websites and different tagging procedures. Distinct strategies were therefore devised for searching and collecting CC-related articles. For DN, we used the website’s own search engine, sorted by date, with the terms “alterações climáticas” (“climate change”) and “aquecimento global” (“global warming”), with additional articles obtained by searching manually using the tags with the same terms. For Observador, we used the date-sorted section “alterações climáticas” (“climate change”) of the newspaper. Due to the high number of CC-related articles located in Observador, a systemic sampling method was developed, with a sampling interval of four (a number produced randomly in the specified range of numbers 1 through 10 using Random.org’s random number generator).

In the first step, we collected all CC-related articles and their respective comments. In the second step, we defined criteria for retaining substantive comments for detailed analysis. Comments were considered substantive according to two conditions: (1) using grammatically well-crafted sentences conveying clear arguments developed enough for categorization and (2) being connected with the press-piece commented on.

### Codification of articles and comments

All comments retained were coded with the neutralization categories of the US CCCM study (McKie, 2018): Denial of Human Responsibility, Denial of Injury, Denial of Victim, Condemnation of the Condemner, and Appeal to Higher Loyalties. We detected in the comments the presence of the argument that CC is not happening, and so we added that category. When comments used more than one neutralization technique, the predominant one was chosen for coding. In the second step, comments were coded according to their functions, that is, the meaning barriers they predominantly employed: Avoiding, Delegitimizing, and Limiting (Gillespie, 2020).

Once the comments to be retained were identified, the articles to which they were responding were coded according to three aspects: (1) *Position toward CC*: Affirming (explicitly supporting dominant CC discourses); Neutral (neither reproducing nor contesting the majority position, providing authorship to CC-related affirmations) or Skepticizing (explicitly contesting/opposing the dominant CC position with or without authorship); (3) *main topic discussed* (see Supplemental Material for a view of topics). The first author coded the articles and comments, while the second author revised all codes. Then, the few differences that emerged were discussed and resolved by consensus.

## 5. Results

### Presence of CC minority positions in articles and comments

As shown in Table 1, 254 articles were collected from DN. Of these, 71 articles had comments, and these amounted to a total of 428, with a total of 103 comments with minority arguments. Of these, 28 (27%) obeyed our retaining criteria. In Observador, 127 articles were generated and inspected. Of these, 99 articles had comments, with a total of 1201 comments, and those expressing CC minority positions amounted to a total of 348. Of these 348 comments, 71 (20%) obeyed the retaining criteria. Therefore, on the whole, 99 substantive comments were retained. Of these 99 comments, 83 come from different accounts (26 different accounts in DN and 57 in Observador).

**Table 1.** Number of climate change articles and their comments per newspaper.

Newspaper	CC articles	CC articles w/comments	Comments	Comments w/CC minority positions	Substantive comments w/CC minority positions
DN	254	71	428	103 (24%^a^)	28 (27%^b^)
Observador	127	99	1201	348 (30%^a^)	71 (20%^b^)
Total	381	170	1629	451 (28%^a^)	99 (22%^b^)

a Percentages express the number of comments with CC minority positions in relation to the total number of comments.

b Percentages express the number of substantive comments with CC minority positions in relation to the total number of comments with CC minority positions.

Therefore, regarding our first research question, *CC minority messaging was clearly present in online readers’ comments*—in the right-leaning newspaper Observador slightly more, when compared with DN (30% vs 24% of all comments), in line with the literature (Schmid-Petri, 2017). Moreover, substantive comments making well-articulated minority CC arguments available for others to learn and reproduce were found to be a relevant proportion of all minority comments—22% overall.

Regarding now how the CC press articles are received: as shown in Table 2, the 23 CC-affirming articles (10 in DN and 13 in Observador) received nearly half (46%) of all substantive comments; neutral articles, in turn, gathered 37% of them. This means that a lot of discursive effort was put into receiving CC-affirming articles and neutral ones in an oppositional way (Hall, 1993). Moreover, the five Observador articles with CC minority positions received 17% of the substantive comments, which also shows that effort was put into supporting (the few) CC-skeptical pieces.

**Table 2.** Comments retained by articles’ position.

		DN			Observador			Total	
		No. of articles	No. of comments	%	No. of articles	No. of comments	%	*n*	%
Position of article toward CC	Affirming	10	15	54	13	30	42	45	**46**
	Neutral	5	13	46	7	24	34	37	37
	Skepticizing	-	-	-	**5**	17	24	17	17
Total		15	28		25	71		99	

As the Supplemental Material shows, regarding the topics of the articles, Greta Thunberg and climate-strike issues were of particular interest to commentators, especially in Observador. Pieces highlighting calls to action by scientists, politicians (e.g., the UN Secretary-General), and environmental activists in general—or what right-wing populist discourses often call the “elites”—also resulted in numerous substantive comments.

### Making sense of CC: Neutralization strategies and meaning barriers

Table 3 displays the results regarding the Neutralization techniques employed. It shows how the dominant technique used is Condemnation of the Condemner, with more than half the comments (52%) falling in this category.

**Table 3.** Frequency of climate change techniques of neutralization in comments retained.

Technique of neutralization	DN		Observador		Total	
	*N*	%	*n*	%	*N*	%
**Denial of Human Responsibility**	**5**	**18**	**16**	**23**	**21**	**21**
Denial of Injury	0	0	1	1	1	1
Denial of Victim	0	0	2	3	2	2
**Condemnation of the Condemner**	**12**	**43**	**39**	**55**	**51**	**52**
Appeal to Higher Loyalties	3	11	7	10	10	10
**CC is Not Happening**	**8**	**28**	**6**	**8**	**14**	**14**
Total	28		71		99	

The second most-used category is Denial of Human Responsibility, with 21% of comments; and the third most-used (14%) is the one claiming that CC is not happening. These are two categories that both express *epistemic* skepticism (Capstick and Pidgeon, 2014), and together they constitute 35% of all substantive comments, demonstrating a very expressive presence of simple CC denial in these press comments.

The fourth category is Appeal to Higher Loyalties, which is detected in 10% of the comments. The other categories are residual—no significant discursive effort was directed at denying injuries (harms) or victims, with comments concentrating on attacking those who make CC a salient issue, defending it causes harms that need to be addressed (the condemners).

Table 4 presents the results obtained from examining how commentators used the three types of meaning barriers.

**Table 4.** Frequency of meaning barriers in comments retained.

Meaning barrier	DN		Observador		Total	
	*n*	%	*n*	%	*n*	%
Avoiding	5	18	22	31	27	27
**Delegitimizing**	**18**	**64**	**40**	**56**	**58**	**59**
Limiting	5	18	9	13	14	14
Total	28		71		99	

Findings show that the most-used barrier in both newspapers is delegitimizing (59%), showing the comments retained were highly focused on condemning the defenders/protagonists of CC by delegitimizing them. Avoiding was also often used (27%). Comments using the barrier of limiting were the least frequent ones (14.14%). This discursive configuration shows how well-articulated comments are more oriented to ignoring or stereotyping the Other—that is, oriented to raising controversy—than searching for a coming together of positions around a negotiation of loyalties (i.e., goals and values) for reaching an eventual consensus.

Finally, the joint analysis of the two types of categorization allows looking at the arguments/categories and their functions in an integrated way.

From a theoretical perspective, the comments Condemning the Condemner should carry stereotyping and stigmatizing arguments working to delegitimize the others and their ideas, and in fact most of these comments (37) are used for this function, as shown in table 5. Appeal to Higher Loyalties works also, as expected, for Limiting. For the other categories, the correspondences are less clear, showing that, despite the general pattern following what was expected, the same arguments can be used with different functions. We thus now turn to illustrate how the same categories can be used with different functions.

**Table 5.** Co-occurrence of meaning barriers and techniques of neutralization.

	Avoiding	Delegitimizing	Limiting	Total
	N	n	n	n
Denial of Human Responsibility	6	**12**	3	21
Denial of Injury	0	1	0	1
Denial of Victim	2	0	0	2
Condemnation of the Condemner	**12**	**37**	2	51
Appeal to Higher Loyalties	0	1	**9**	10
CC is Not Happening	7	7	0	14
Total	27	58	14	99

Extract 1 illustrates how Denial of Human Responsibility for CC is used for *avoiding*—it is simply focused on excluding the dominant meanings from even being considered:

Extract 1—Denial of Human Responsibility *for* Avoiding

*The climate was already on a gradual warming trajectory before humans started to have a significant influence*. Furthermore, abrupt changes in the average (temperatures) have occurred before and not that long ago. We had a mini-ice age in the 14th century that lasted until the 18th century. We had a rapid warming after that. (to article 9, DN)

The argument is anchored in descriptions of past CC working as a barrier to avoid the present majority/dominant epistemic assumptions. Although it does not deny the existence of CC, it fully dismisses humanity’s influence on it, working to suppress any need for more attention, concern or action.

Other comments take a different, less drastic, strategy—simultaneously denying human responsibility for CC and orienting to delegitimize CC majority positions. Extract 2 illustrates one such case:

Extract 2—Denial of Human Responsibility *for* Delegitimizing

The point here is that reducing emissions has an immediate effect on increasing air quality—which shows that *the problem is less structural than has been conveyed*.

Of course, once this is understood, the whole narrative of climate cataclysm (which is different from environmental cataclysm, produced mainly by trash and other waste in soils, aquifers, and oceans) falls apart. That is, *the atmosphere regenerates very quickly, and this does not fit the beliefs of school-kids and their instructors*. (to article 27, Observador)

COVID-19 is here entering the CC debate in reaction to an opinion piece describing the drop in emissions caused by reduced human activity during the pandemic, being called upon to demonstrate that such activity produces only quickly neutralizable effects, not structural ones, thus delegitimizing activists like those of Fridays for Future (*school-kids and their instructors)* and the *whole narrative of climate cataclysm.* The comment uses the emission reductions of the pandemic to also specify that CC and environmental problems (waste in soils, aquifers, and oceans) are different issues, presenting its argumentation as reasonable, and delegitimizing defenders of CC for failing to recognize this.

Let us look now at how *Condemnation of the Condemner* is used with different functions. Sometimes arguments of this kind are used to avoid, as in Extract 3:

Extract 3—Condemnation of the Condemner *for* Avoiding

The anti-CO2 ideological blindness, as if it were pollution, leads to this. *Actually, it’s just another excuse for the “central planning” that is supposed to save us from the end of the world. Communist tricks.* (to article 30, Observador)

This comment expresses blatant disagreement with the idea that CO_2_ is a bad thing—accusing those who agree with it of “ideological blindness.” This is a disagreement that fully avoids dealing even with the most central majority meaning, the deleterious effects of CO_2_. Here the function played by the word “ideological,” used as a curse word, is that of portraying (unspecified) others as blinded by their (defective) convictions (or ideology) using CC advocacy as “an excuse” to accomplish “communist tricks.” As such, the others’ ideas are again fully avoided by being fully denied, like in Extract 1. But here the meaning categories of the far-right and populist discourse (“communist tricks,” “central planning”) are also mobilized, again revealing their link with CC minority positions (Küppers, 2024; Lockwood, 2018; Vihma et al., 2021).

Comments can also be highly engaged with delegitimizing highly specific CC actors. This is illustrated by Extract 4:

Extract 4—Condemnation of the Condemner *for* Delegitimizing

*These “journalists” or rather activists are well trained. A few more little taxes are coming down on the people while the elite who preach climate disaster ride private jets, but that can’t be said.* CNN was already exposed last week by one of its directors, who assumed what many people know, that they do propaganda and not journalism; others will follow suit. (to article 36, Observador)

Here, the acceptance of the existence of CC remains unspecified, but it is clear that the comment attacks the elite who preach climate disaster and do not act accordingly. It also portrays journalists aligned with CC-affirming discourses in a negative, stereotyped way, depicting them as propagandists or activists, that is, as breaching the code of the profession, so, as illegitimately occupying the role of journalists. Mobilized to give credibility to this negative depiction is (an un-identified) CNN director, credited with having revealed “the truth”—that CNN does propaganda, not journalism—on one (un-identified) occasion. Thus, what seems to be the mobilization of resources of credibility—a director whose high position gives access to inside information—is coupled with a blurring of who the person and the occasion are. To be noted is the attention given to CNN in this comment in a Portuguese newspaper, indicating attention to the worldwide CC debate.

Finally, commentators using this technique can condemn the condemner by showcasing concerns that are viewed as more important than those that CC advocates defend. Extract 5 shows how this strategy, and the barrier of limiting can co-exist:

Extract 5—Condemnation of the Condemner *for* Limiting

(. . .) these young people are being exploited by marxist-leninists, disguised as “greens” and “environmentalists,” who are much more interested in causing problems for businessmen (for “capital,” as they say) than in solving any eventual environmental problems. *It has been clearly identified that the origin of all environmental problems is the galloping population growth in Africa and Asia, which these activists do not have the courage to speak out against.* (to article 4, DN)

In this extract, CC is portrayed as a worthy cause currently in the wrong hands—(undefined) CC actors (greens and environmentalists) are accused of being Marxist-Leninists in disguise, interested not in solving environmental problems but in causing problems for businessmen and capitalism and accused of exploiting the young under false pretenses. This opens space for affirming that the real problems, or environmental priorities, are elsewhere (and far away)—the galloping population growth in Africa and Asia—but no one has courage to reveal them. Thus, these arguments implicitly work to present their defender as credibly recognizing the existence of environmental problems, and justifying the need to limit CC policies by calling for policy efforts to focus on other more pressing issues.

Moving now to the technique of appealing to higher loyalties, there are clear links with the meaning barrier Limiting, with nine out of the 10 retained comments that used Appeals to Higher Loyalties resorting to this barrier. Extract 6 illustrates this combination:

Extract 6—Appeal to Higher Loyalties *for* Limiting

Throughout the history of the world there have been areas that were once submerged and others that were not and still are. The same goes for the climate. Who says so are the scientists of the UN and others. *If the attention they give to climate change and ocean levels* (all to collect more taxes and impose ideological dictatorship) *were put at the service of combating plastic, rubbish and excessive consumption we would all win*. (to article 17, Observador)

The argument that CC, while real, is essentially natural in its causes is here complemented by a prioritization of other values—such as action against excessive human pollution and consumption. CC action is again depicted in a populist vocabulary, as meant to “collect more taxes and impose ideological dictatorship,” and climate science and climate policy are disconnected. The commentator distinguishes between what “scientists of the UN and others” say (“there have been areas that were once submerged and others that were not and still are”), and what CC advocates are trying to do (impose ideological dictatorship). The latter’s ideas are limited by being portrayed as having nothing to do with the preservation of the environment. So, interestingly, epistemic authority is called upon to support response skepticism.

Finally, there are still comments fully denying CC, as this last extract shows:

Extract 7—CC is Not Happening *for* Avoiding

*They’ve been carrying on with the global warming crap for decades, always repeating the same ridiculous catastrophic predictions that are always coming. There’s no patience.* (to article 34, Observador)

The comment here is expressing full epistemic skepticism—simply working to avoid (engage with) the issue in any manner.

## 6. Conclusion

Discourses denying the anthropogenic nature of CC, and the need for climate action and policy, are today minority ones in the worldwide CC debate. However, that does not mean that they do not impact it, nor is it impossible that they may grow in popularity. There are indeed ongoing, concerted efforts in formal forums to keep them alive, and even make them more widespread—not only in the United States but also in EU countries (Almiron et al., 2020), from conservative foundations, think-tanks (McKie, 2018), and from the far-right and right-wing populist parties (Forchtner and Lubarda, 2023; Lockwood, 2018). Since the press is today also an important outlet for online readers’ comments (Domínguez et al., 2016), these are now a crucial resource for investigating how everyday minority understandings of CC meet CC press coverage. It is important to study their alignment with those of formal forums, and how they circulate minority meanings, making them available to be learned and further reproduced.

In this study, we thus looked at (1) the extent to which, in two Portuguese mainstream newspapers of different ideological orientations, but of similar and high circulation, readers’ comments to CC press articles kept CC minority meanings alive and circulating, and to what extent those comments were both numerous and well-articulated. Then, we (2) used the neutralization technique categories used to study formal US CC Counter Movement discourses and applied it to the study of the 99 well-articulated comments found, together with a systematization of the barriers that can be raised in discourse against the worldviews of others (Castro and Santos, 2020; Gillespie, 2008, 2020). In that sense, the goals were to obtain a nuanced picture of the everyday discursive skeptic effort in the Portuguese mainstream press, adding detail to the broader categories of epistemic and response skepticism (Capstick and Pidgeon, 2014; Forchtner and Lubarda, 2023) and shedding light on their functions: for example, to question the existence of CC; to undermine proponents of social change through negative, stereotyping portrayals; to criticize CC policies by proposing other priorities.

Findings show that readers’ comments to CC pieces were numerous in both newspapers—with those expressing CC minority ideas being a substantial part of the set (28%). They reveal too that minority comments were only slightly more numerous in the right-leaning newspaper (30% vs 24%), showing that while conservative newspapers may provide more of a forum for such positions (Schmid-Petri, 2017) other newspapers are also home to them.

Findings also show that a good percentage of these minority comments (22%) was substantive, using well-articulated arguments, not only mono or dissyllabic invectives. To be noted is the fact that nearly half of all well-articulated comments (46%) were directed to attack CC articles aligned with majority discourses, and 37% to neutral ones—suggesting that commentators are putting a lot of effort into opposing the dominant view. However, some well-articulated comments (17%) were also directed to the few articles with CC minority views, revealing an effort to also support such stances.

Finally, the findings obtained for the Neutralization strategies indicate that while only 14% of well-articulated comments directly deny that CC is happening 21% defend the position that humans have no responsibility for it, that is, that CC is not necessarily anthropogenic. Therefore, if we join these two categories, we have 35% of these comments working for advancing some form of *epistemic* skepticism about CC—which is undoubtfully a high percentage.

Nevertheless, the most significant part of the discursive effort with these techniques is placed on condemning proponents and protagonists of climate action (*Condemning the Condemners*), attacking the CC “elites”: journalists, activists, national and international policy-makers. In this, the comments here analyzed are reproducing the pattern found for US conservative foundations and think-tanks (McKie, 2018) and EU right-wing parties. Moreover, finding show how the dominant barrier mobilized to “condemn” CC actors is delegitimization—the use of negative, stereotyped, stigmatizing person-focused arguments more than idea-focused ones. These stereotypes are often constructed by mobilizing a right-wing populist vocabulary, depicting CC “elites” as Marxists and centralists in disguise, corroborating studies linking populism and CC skeptic narratives in EU countries (Küppers, 2024; Lockwood, 2018; Vihma et al., 2021). Also to be remarked is that the comments are not very focused on arguing for limiting CC policies by appeals to other priorities and concessive “yes. . .but” discourses (Uzelgun et al., 2016)—only 10% of them use the category Appeal to Higher Loyalties, and only 14% use the barrier Limiting. Mobilizing negative emotions, they are more oriented toward controversy and discredit, than toward moving social efforts to negotiation for consensus around relevant issues (Uzelgun et al., 2016). And in this, too, they are consistent with other forums.

In sum, our findings shed further light on everyday minority public understanding of CC in Portugal, and how it connects to the wider CC debate, in three ways. First, they show how in Portugal readers’ comments in the mainstream press play an important role in the reproduction and circulation of CC minority ideas—showing that not just comments in tabloids do this work. Second, they provide valuable indications of how the everyday CC minority messaging in the press in Portugal has moved beyond insisting on outright denial—simple epistemic skepticism—and into very negative, person-stigmatizing contestation of CC actors, presented as “elites” in populist-charged vocabularies. This is rather coherent with United States and European far-right parties and organizations (Forchtner and Lubarda, 2023), reinforcing the idea of a growing European CC minority effort also being waged in similar ways in everyday forums—and re-enforcing the idea that mobility in meaning is an indication of mobility in power relations. Yet it is important to note also a more surprising finding: the comments continue to offer the Portuguese public an insistent contestation of the anthropogenic nature of CC.

One limitation of this study in this regard is that it is not possible to determine the number of unique commentators represented in the 99 well-articulated comments analyzed, which would help in better understanding the extent of the Portuguese network engaged in pushing this content into the public sphere. We know that 83 of the 99 comments come from different accounts, but it is possible for commentators to have multiple accounts, and we have no form of tracing this. Nevertheless, from the meaning-making perspective adopted here, the most relevant fact is that the content is indeed placed *out there* for others to learn and reproduce it, sustaining and/or amplifying the set of updated arguments available for waging the CC battle of ideas from minority trenches, maintaining their meanings “quarantined” for change.

Third, the article pays attention to how meaning barriers are employed from minority positions to contest highly dominant meanings, and these findings contribute to extending the literature in this scarcely studied direction. The study identifies a minority communication with predominantly de-legitimizing functions consistently oriented to confrontation, suggesting that when certain meanings become highly dominant in a debate, more than simple *avoidance* through denial is needed, and delegitimizing may increase. Finally, also worthy of notice is here that these well-articulated arguments are not at all focused on blaming victims, even though blaming the victim is a much-employed neutralization technique in various areas (Bandura, 2007; Kaptein and Van Helvoort, 2019). Maybe this is due to the fact that harm is also insufficiently recognized, so victims cannot get recognition, and the discursive effort is instead, as we saw, placed on attacking those offering recognition to the existence of harms and their victims.

More research is however needed to clarify this, as well as other potential differences in the patterns of use of these barriers from majority and minority positions, helping understand if there might exist different predominant barriers in use from each position.

A further limitation of this study is that the discursive strategies and barriers are not mutually exclusive in all the comments. Therefore, studies with a more detailed reconstruction of their arguments—for example, identifying through Argumentation theory (Uzelgun et al., 2016) the different paths each of the barriers may take—are needed.

Nevertheless, the confrontational and delegitimizing pattern found suggests that it is essential for decision-makers and other CC actors to develop strategies that bring those with minority positions closer to the negotiation of ideas and policy priorities, and ultimately finding areas of consensus. In the future, it would be relevant to analyze how the discursive contents and functions of these CC minority discourses evolve over time in readers’ comments, gaining further insight into the effects of the international circulation of discourses and their links to everyday discourse in Portugal. Moreover, it is crucial to look at the programmatic policies of the new far-right and liberal political parties with parliamentary representation, assessing their alignment with these comments and the role that they are playing in the CC debate as they rise in popularity.

## Supplemental Material

sj-docx-1-pus-10.1177_09636625241254505 – Supplemental material for The climate battles of ideas: Minority discourses in readers’ comments to climate change articles in the Portuguese press Supplemental material, sj-docx-1-pus-10.1177_09636625241254505 for The climate battles of ideas: Minority discourses in readers’ comments to climate change articles in the Portuguese press by Nuno Monteiro Ramos and Paula Castro in Public Understanding of Science
