# Supplementary material for: The climate battles of ideas: Minority discourses in readers’ comments to climate change articles in the Portuguese press
Source: Public Underst Sci. 2024 Jun 12;34(1):59–75. doi: 10.1177/09636625241254505 (PMC11673290; doi:10.1177/09636625241254505)
Supplement: sj-docx-1-pus-10.1177_09636625241254505 – Supplemental material for The climate battles of ideas: Minority discourses in readers’ comments to climate change articles in the Portuguese press [file sj-docx-1-pus-10.1177_09636625241254505.docx]

**Title**

The climate *battles of ideas*: minority discourses in readers’ comments to climate change articles in the Portuguese press

**Authors**

Nuno M. Monteiro Ramos^a^, Paula Castro^b^

**Affiliations**

^a^Instituto Universitário de Lisboa (ISCTE-IUL), Lisboa, Portugal

^b^Instituto Universitário de Lisboa (ISCTE-IUL), CIS-IUL, Lisboa, Portugal

**Supplemental material**

Topics discussed in the DN and Observador articles that received substantive comments

|  | Article n. º | Title of the article  (in English) | Publication date | Type of article | Topic discussed | Position  towards CC | N. º of comments | N. º of comments retained | URL |
| --- | --- | --- | --- | --- | --- | --- | --- | --- | --- |
| **DN** | 1 | Mediterranean warming is 20% faster  than the global average | 10/10/2019 | News story | Effects of CC | Affirming | 7 | 1 | <https://www.dn.pt/vida-e-futuro/aquecimento-da-temperatura-no-mediterraneo-e-20-mais-rapido-que-media-do-planeta-11391100.html> |
|  | 2 | Interactive map shows areas of Portugal at risk from rising oceans | 29/10/2019 | News story | Effects of CC | Affirming | 20 | 3 | <https://www.dn.pt/vida-e-futuro/em-31-anos-a-subida-do-nivel-dos-oceanos-pora-em-risco-300-milhoes-de-pessoas-11459788.html> |
|  | 3 | Lisbon will plant 20 thousand trees  and prepare for climate change | 29/11/2019 | News story | Climate action | Neutral | 4 | 1 | <https://www.dn.pt/cidades/lisboa-vai-plantar-20-mil-arvores-e-preparar-se-para-as-alteracoes-climaticas--11567016.html> |
|  | 4 | Climate Strike. "We won't give up until we win | 29/11/2019 | News story | Climate strike | Affirming | 21 | 2 | <https://www.dn.pt/vida-e-futuro/greve-climatica-nos-nao-vamos-desistir-ate-ganhar-11564124.html> |
|  | 5 | António Guterres: "We must stop our war  against nature | 01/12/2019 | News story | Call to action on CC | Neutral | 3 | 1 | <https://www.dn.pt/vida-e-futuro/alteracoes-climaticas-guterres-apela-ao-aumento-da-vontade-politica-de-lideres-mundiais--11572601.html> |
|  | 6 | Victoria Falls reduced to a trickle due to  extreme drought | 07/12/2019 | News story | Effects of CC | Affirming | 10 | 2 | <https://www.dn.pt/mundo/cataratas-de-vitoria-reduzidas-a-fio-de-agua-devido-a-seca-extrema-11593499.html> |
|  | 7 | The main conclusions of the climate summit that disappointed Guterres | 15/12/2019 | News story | Climate summit | Affirming | 11 | 1 | <https://www.dn.pt/vida-e-futuro/as-principais-conclusoes-da-cimeira-do-clima-que-desiludiu-guterres-11620414.html> |
|  | 8 | Youtube directs millions of users to climate disinformation | 17/01/2020 | News story | Fake news on CC | Neutral | 1 | 1 | <https://www.dn.pt/vida-e-futuro/youtube-encaminha-milhoes-de-utilizadores-para-desinformacao-climatica-11719089.html> |
|  | 9 | At the age of 19, Naomi is the anti-Greta.  Her word is already spreading around the world | 02/03/2020 | News story | Naomi Seibt | Neutral | 77 | 7 | <https://www.dn.pt/mundo/com-19-anos-naomi-assume-se-como-a-anti-greta-a-sua-palavra-ja-corre-o-mundo-11875656.html> |
|  | 10 | "Almost 100% of deforestation in Brazil is criminal," says scientist | 08/10/2020 | News story | Illegal  deforestation | Affirming | 11 | 1 | <https://www.dn.pt/edicao-do-dia/08-out-2020/quase-100-do-desmatamento-no-brasil-e-criminoso-diz-cientista-12887824.html> |
|  | 11 | Portugal and Spain will "bake" with average temperature increase | 18/11/2020 | News story | Effects of CC | Affirming | 23 | 1 | <https://www.dn.pt/vida-e-futuro/portugal-e-espanha-vao-assar-com-aumento-medio-da-temperatura-13049604.html> |
|  | 12 | Human Rights Court welcomes action from Portuguese youth and orders 33 countries to respond | 30/11/2020 | News story | Climate action | Affirming | 4 | 1 | <https://www.dn.pt/mundo/tribunal-dos-direitos-humanos-da-luz-verde-a-processo-movido-por-jovens-portugueses-13090315.html> |
|  | 13 | It has already happened in history. If Greenland's ice melts coastal cities will be under water | 16/03/2021 | News story | Effects of CC | Affirming | 4 | 2 | <https://www.dn.pt/ciencia/ja-aconteceu-na-historia-se-o-gelo-da-gronelandia-derreter-as-cidades-costeiras-ficam-submersas-13466196.html> |
|  | 14 | Guterres says the world is "on the edge of the abyss" | 22/04/2021 | News story | Call to action on CC | Neutral | 12 | 3 | <https://www.dn.pt/internacional/guterres-pede-coligacao-global-comprometida-com-emissoes-zero-em-2050--13600668.html> |
|  | 15 | The Brazilian Amazon already emits more carbon than it absorbs | 30/04/2021 | News story | Effects of CC | Affirming | 6 | 1 | <https://www.dn.pt/internacional/amazonia-brasileira-ja-emite-mais-carbono-do-que-absorve-13632601.html> |

|  | Article n. º | Title of the article (in English) | Publication date | Type of article | Topic discussed | Position  towards CC | N.º of comments | N.º of comments retained | URL |
| --- | --- | --- | --- | --- | --- | --- | --- | --- | --- |
| **Observador** | 16 | Scientists send "strong message" on climate change to world leaders | 24/06/2019 | News story | Call to action on CC | Neutral | 17 | 2 | <https://observador.pt/2019/06/24/cientistas-enviam-mensagem-forte-sobre-alteracoes-climaticas-aos-lideres-mundiais/> |
|  | 17 | Ocean levels could rise with consequences for more than a billion people, new UN study warns | 25/09/2019 | News story | Effects of CC | Neutral | 61 | 13 | <https://observador.pt/2019/09/25/nivel-dos-oceanos-pode-subir-com-consequencias-para-mais-de-mil-milhoes-de-pessoas-alerta-novo-estudo-da-onu/> |
|  | 18 | Greta among the Doctors | 01/10/2019 | Opinion piece | Greta Thunberg | Skepticizing | 54 | 1 | <https://observador.pt/opiniao/greta-entre-os-doutores/> |
|  | 19 | Dear Greta, this is my message to you… | 24/10/2019 | Opinion piece | Greta Thunberg | Skepticizing | 143 | 9 | <https://observador.pt/opiniao/querida-greta-esta-e-a-minha-mensagem-para-ti/> |
|  | 20 | Greta Thunberg "is an idiot," says Jeremy  Clarkson | 28/11/2019 | News story | Greta Thunberg | Neutral | 66 | 3 | <https://observador.pt/2019/11/28/greta-thunberg-e-uma-idiota-diz-jeremy-clarkson/> |
|  | 21 | Guterres calls for increased political will from world leaders to fight climate change | 01/12/2019 | News story | Call to action on CC | Neutral | 35 | 1 | <https://observador.pt/2019/12/01/guterres-apela-ao-aumento-da-vontade-politica-dos-lideres-mundiais-na-luta-contra-alteracoes-climaticas/> |
|  | 22 | Paris Agreement looming and a collective failure. Can a summit avoid "climate catastrophe"? | 02/12/2019 | Opinion piece | Climate summit | Affirming | 73 | 3 | <https://observador.pt/especiais/acordo-de-paris-a-espreita-e-um-falhanco-coletivo-pode-uma-cimeira-evitar-a-catastrofe-climatica/> |
|  | 23 | When all prophets become dangerous.  Including Greta Thunberg | 04/12/2019 | Opinion piece | Greta Thunberg | Skepticizing | 108 | 4 | <https://observador.pt/especiais/quando-todos-os-profetas-se-tornam-perigosos-incluindo-greta-thunberg/> |
|  | 24 | Little Greta gave me something to believe in | 07/12/2019 | Opinion piece | Greta Thunberg | Skepticizing | 17 | 2 | <https://observador.pt/opiniao/a-pequena-greta-deu-me-alguma-coisa-em-que-acreditar/> |
|  | 25 | Antarctica recorded temperature above 20ºC? It may not be quite like that after all | 20/02/2020 | News story | Effects of CC | Neutral | 5 | 2 | <https://observador.pt/2020/02/20/antartida-registou-temperatura-acima-dos-20oc-afinal-pode-nao-ser-bem-assim/> |
|  | 26 | Planet's temperature may stabilize at values of three million years ago, says expert | 26/02/2020 | News story | Effects of CC | Skepticizing | 5 | 1 | <https://observador.pt/2020/02/26/temperatura-do-planeta-pode-estabilizar-nos-valores-de-ha-tres-milhoes-de-anos-diz-especialista/> |
|  | 27 | No, the pandemic is not good for the environment. But it may leave clues for a sustainable future | 01/04/2020 | Opinion piece | Call to action on CC | Affirming | 40 | 6 | <https://observador.pt/especiais/nao-a-pandemia-nao-e-boa-para-o-ambiente-mas-pode-deixar-pistas-para-um-futuro-sustentavel/> |
|  | 28 | Government announces 1,3 million euros to assess vulnerability to climate change | 07/09/2020 | News story | Climate action | Neutral | 3 | 2 | <https://observador.pt/2020/09/07/governo-anuncia-13-milhoes-de-euros-para-avaliar-vulnerabilidade-as-alteracoes-climaticas/> |
|  | 29 | 2021: to start changing one's life | 27/12/2020 | Opinion piece | Call to action on CC | Affirming | 7 | 1 | <https://observador.pt/opiniao/2021-comecar-a-mudar-de-vida/> |
|  | 30 | Leaving Portugal in the dark and cold | 09/01/2021 | Opinion piece | Energy Transition | Affirming | 10 | 1 | <https://observador.pt/opiniao/deixar-portugal-as-escuras-e-ao-frio/> |
|  | 31 | Why is this the coldest winter of the decade in Portugal and in Spain it is colder than in Norway? | 14/01/2021 | News story | Weather | Affirming | 8 | 3 | <https://observador.pt/2021/01/14/porque-e-que-este-e-o-inverno-mais-frio-da-decada-e-em-portugal-esta-mais-frio-em-espanha-do-que-na-noruega/> |
|  | 32 | It snowed in the Sahara: the fourth time in the last 42 years | 27/01/2021 | News story | Effects of CC | Affirming | 5 | 1 | <https://observador.pt/2021/01/27/nevou-no-sahara-e-a-quarta-vez-nos-ultimos-42-anos/> |
|  | 33 | Meeting the Paris Agreement on climate change could save millions of lives, study finds | 10/02/2021 | News story | Call to action on CC | Neutral | 2 | 1 | <https://observador.pt/2021/02/10/cumprir-acordo-de-paris-sobre-alteracoes-climaticas-poderia-salvar-milhoes-de-vidas-conclui-estudo/> |
|  | 34 | Bill Gates: "Climate change is harder to solve than a pandemic" and effects could "be much worse" | 15/02/2021 | News story | Call to action on CC | Affirming | 29 | 6 | <https://observador.pt/2021/02/15/bill-gates-as-alteracoes-climaticas-sao-mais-dificeis-de-resolver-do-que-uma-pandemia-e-efeitos-podem-ser-muito-piores/> |
|  | 35 | Bill Gates' plan for the climate crisis - that will kill five times more than the pandemic | 16/02/2021 | Feature | Effects of CC | Affirming | 10 | 1 | <https://observador.pt/especiais/o-plano-de-bill-gates-para-a-crise-climatica-que-vai-matar-cinco-vezes-mais-que-a-pandemia/> |
|  | 36 | Europe experienced warmest year in 2020, Siberian Arctic at 6º C above average | 22/04/2021 | News story | Effects of CC | Affirming | 6 | 3 | <https://observador.pt/2021/04/22/europa-viveu-ano-mais-quente-em-2020-aritco-siberiano-com-6o-c-acima-da-media/> |
|  | 37 | EU Presidency: Ministers discuss impact of climate change on water management | 23/04/2021 | News story | Effects of CC | Affirming | 2 | 1 | <https://observador.pt/2021/04/23/presidencia-da-ue-ministros-discutem-impacto-das-alteracoes-climaticas-na-gestao-da-agua/> |
|  | 38 | With no beaches and with 50ºC in Beja. How can the country "most vulnerable in Europe" to climate change be in 2071? | 05/05/2021 | Feature | Effects of CC | Affirming | 8 | 1 | <https://observador.pt/especiais/sem-praias-e-com-50oc-em-beja-como-pode-ser-em-2071-o-pais-mais-vulneravel-da-europa-as-alteracoes-climaticas/> |
|  | 39 | Frans Timmermans. Keeping temperature increase below 2ºC "will be very difficult, but we have to try". | 02/06/2021 | Interview | Call to action on CC | Affirming | 4 | 2 | <https://observador.pt/especiais/frans-timmermans-manter-aumento-de-temperatura-abaixo-dos-2oc-vai-ser-muito-dificil-mas-temos-de-tentar/> |
|  | 40 | Lithium and the "fable" of decarbonization | 19/06/2021 | Opinion piece | Effects of CC | Affirming | 9 | 1 | <https://observador.pt/opiniao/o-litio-e-a-fabula-da-descarbonizacao/> |
